# Hints for Probationer Nurses

**Published:** 1887-12-03

**Authors:** 


### Hints for Probationer Nurses.

The following instructions will be found useful to others besides the probationers for whom they were written. They are worthy of careful study and attention, and cannot fail to prove of interest to many:—

1. Pare the finger-nails close; keep them, as well as the fingers and hands, scrupulously clean.

2. Anything which soils the fingers is a possible source of contagion to others and to self. No one who poisons her finger is fit to be a nurse. If she cannot take care of her own cleanliness, how can she take care of her patients'?

3. A hang-nail, or crack, or scratch, or pin-puncture, is likely to prove a poison-nest to others or to self, even more than an open wound or sore.

4. Such poison-nests must be rendered harmless by first washing with pure water; secondly, by the application of styptic colloid; thirdly, by being covered with an indiarubber finger-stall.

5. Immediately *before* beginning any dressing, and in every case *after* a nurse has *touched* her patient in dressing wounds, rubbing in applications, administering enemata, internal syringing, washing out eyes, ears, nose, mouth, the hands are to be dipped in watery solution of carbolic acid, 1 to 80, and then to be washed and nails cleaned carefully with carbolic soap. Dressing forceps, or syringe, or whatever is used, to be dipped in solution of carbolic acid (1 to 80) *before* use as well as *after*. The teeth and joints of the dressing forceps must also be brushed clean.

6. Soiled dressings should be removed with dressing forceps, and not with fingers; and on no account should adhesive plaster or other adhering dressings be scratched up with finger-nails.

7. With all *internal* cases, keep finger-nails short, fill the same with carbolic soap, and anoint carefully the fingers about to be used with carbolic oil, 1 in 20. Oil the tube or nozzle, &c., to be used for any internal application with carbolic oil, 1 in 20; otherwise the appliances used might convey contagious matter from one patient to another.

8. Always use *two* basins in washing wounds.

9. Catheters, every time they are used, must be cleansed and disinfected, first with a stream of warm water, and then with a stream of watery solution of carbolic acid, 1 to 40. Catheters made of other material than silver should *not* be soaked in carbolic acid solution, as the acid injures varnish and gum. Never *blow down* towards the eye *first* instead of last; for so they are always destroyed by effecting some lodgment at the bottom.

10. After offensive cases, blow the nose and expectorate, and rinse the mouth and throat with Condy and water, 1 to 10; or with two drops of sanitas fluid to two ounces of water.

11. In ophthalmic cases the greatest care requires to be exercised to avoid the poison of the disease, which is eminently contagious. The eye should never be touched by fingers or anything that can by any possibility have come in contact with poisonous matter, and if any symptom of sore eyes is experienced, it should *at once* be reported to the lady superintendent.

12. Cleansed fingers and hands should always be dried on towels which have *not* been used for any other purpose.

13. Look upon cuffs and sleeves and stuff dresses as possible carriers of contagious or infectious matter. The apron and over-sleeves which are worn in the wards should never be worn at meals.

14. Upon discovering any scratch, or crack, or hang-nail, or sore, or wound, report it immediately to the lady superintendent, to whom also immediate application for advice should be made after breathing in an offensive air.

15. The nature of contagion and infection, and the distinctions between disinfectants and antiseptics should be learned at once.

16. Never under any circumstances go on duty in the morning without having taken a meal.

